# Immunomodulatory Nanoparticles Mitigate Macrophage Inflammation via Inhibition of PAMP Interactions and Lactate-Mediated Functional Reprogramming of NF-κB and p38 MAPK

**DOI:** 10.3390/pharmaceutics13111841

**Published:** 2021-11-02

**Authors:** Jackline Joy Martín Lasola, Andrea L. Cottingham, Brianna L. Scotland, Nhu Truong, Charles C. Hong, Paul Shapiro, Ryan M. Pearson

**Affiliations:** 1 Department of Microbiology and Immunology, University of Maryland School of Medicine, 685 W. Baltimore Street, Baltimore, MD 21201, USA; jacklinejoy.lasola@som.umaryland.edu; 2 Department of Pharmaceutical Sciences, University of Maryland School of Pharmacy, 20 N. Pine Street, Baltimore, MD 21201, USA; acottingham@umaryland.edu (A.L.C.); bscotland@umaryland.edu (B.L.S.); ntruong@umaryland.edu (N.T.); pshapiro@rx.umaryland.edu (P.S.); 3 Division of Cardiovascular Medicine, Department of Medicine, University of Maryland School of Medicine, 110 S. Paca Street, Baltimore, MD 21201, USA; charles.hong@som.umaryland.edu; 4 Marlene and Stewart Greenebaum Comprehensive Cancer Center, University of Maryland School of Medicine, 22 S. Greene Street, Baltimore, MD 21201, USA

**Keywords:** inflammation, innate immunity, macrophages, Toll-like receptors, TLR, NF-κB, p38 MAPK, sepsis, poly(lactic acid), PLA, lactate, nanoparticles, microparticles

## Abstract

Inflammation is a key homeostatic process involved in the body’s response to a multitude of disease states including infection, autoimmune disorders, cancer, and other chronic conditions. When the initiating event is poorly controlled, severe inflammation and globally dysregulated immune responses can occur. To address the lack of therapies that efficaciously address the multiple aspects of the dysregulated immune response, we developed cargo-less immunomodulatory nanoparticles (iNPs) comprised of poly(lactic acid) (PLA) with either poly(vinyl alcohol) (PVA) or poly(ethylene-alt-maleic acid) (PEMA) as stabilizing surfactants and investigated the mechanisms by which they exert their inherent anti-inflammatory effects. We identified that iNPs leverage a multimodal mechanism of action by physically interfering with the interactions between pathogen-associated molecular patterns (PAMPs) and bone marrow-derived macrophages (BMMΦs). Additionally, we showed that iNPs mitigate proinflammatory cytokine secretions induced by LPS via a time- and composition-dependent abrogation of NF-κB p65 and p38 MAPK activation. Lastly, inhibition studies were performed to establish the role of a pH-sensing G-protein-coupled receptor, GPR68, on contributing to the activity of iNPs. These data provide evidence for the multimodal mechanism of action of iNPs and establish their potential use as a novel therapeutic for the treatment of severe inflammation.

## 1. Introduction

Severe inflammation is a complex and global multi-step physiological process implicated in the development of a systemic dysregulated immune environment. Using sepsis as an example of severe inflammation, epidemiological data suggests that one-in-five of all global deaths is due to sepsis or sepsis-related causes [1]. However, the standard of care for sepsis has failed to move far beyond antibiotics and supportive care, thus leaving much room for the development of new treatment strategies to improve outcomes. To date, over 100 clinical trials have been conducted for potential therapies, but curative strategies remain elusive [2,3]. Previous attempts to address and manage severe inflammation and sepsis have focused on the development of single molecular agents targeted against specific molecules or aspects of molecular pathways implicated in the development of the severe inflammatory response. Despite these methods often demonstrating outstanding preclinical success, translating these results to viable therapeutics for critically ill patients has been unrealized [4]. It has been hypothesized that these attempts have failed because of the profound clinical heterogeneity of sepsis, the lack of fundamental understandings of the different endotypes of sepsis, and treatments that have been targeted towards only a single molecular pathway, leaving redundant pathways associated with immune activation and a multifaceted immune dysfunction unaddressed [5,6]. Therefore, a significant need exists to develop multimodal therapeutics to address the complexity of immune responses present in severe inflammation and sepsis.

Modulation of the innate immune system using nanoparticles serves as the basis for many new and promising therapies for some of the most prevalent and/or severe diseases [7,8,9]. We recently reviewed the various strategies of nanoparticle-mediated immunomodulation for the treatment of severe inflammation and sepsis [10]. Three mechanisms were proposed by which nanoparticles can be utilized to offset the negative immune mediators of severe inflammation: (1) sequestration of activating pathogen-associated molecular patterns (PAMPs) or proinflammatory cytokines; (2) functional reprogramming of inflammatory immune cell phenotypes; and (3) redirection of inflammatory immune cell trafficking from sites of inflammation.

Our group [11,12] and others [13] have developed cargo-less immunomodulatory nanoparticles (iNPs) that lacked incorporation of small molecules, proteins, or other immunomodulating agents and showed that the physicochemical properties of the nanoparticles were major contributors to the observed therapeutic effects. In our previous studies, antigen presenting cells treated with cargo-less poly(lactic-co-glycolic acid) (PLGA)- and poly(D,L-lactic acid) (PLA)-based iNPs prepared with highly negative zeta potentials could mitigate proinflammatory cytokine secretions such as IL-6 and TNF-α when stimulated with extracellular and intracellular PAMPs, namely Toll-like receptor 4 (TLR4)-targeted lipopolysaccharide (LPS) and TLR9-targeted unmethylated CpG oligodeoxynucleotides (CpG ODN). Furthermore, their immunomodulatory properties translated into a survival benefit in lethal murine LPS-induced endotoxemia models [11]. Initial analysis hinted at a potential role for modulation of NF-κB, IRF1, and STAT1; however, the mechanisms by which iNPs elicit their favorable therapeutic effects remains poorly understood. For these nanoparticle-based strategies to move forward, a greater understanding of the biological effects of these materials and mechanisms by which they exert their immunomodulatory effects is warranted.

Nanoparticles are complex systems and can function through multiple mechanisms where each component involved in its production (i.e., stabilizing surfactant and polymer composition) can potentially alter cellular and inflammatory mediator interactions including rate of uptake, trafficking, rate of degradation and degradation products, etc. Stabilizing surfactants such as poly(vinyl alcohol) (PVA) and poly(ethylene-alt-maleic acid) (PEMA) are ideal for testing the impact of surface characteristics on nano-bio interactions given the variability in zeta potentials and surface chemistry while allowing for control of iNP size. PLA is ideal for understanding the role of the polymer composition and further use in nanoparticle development due to its Food and Drug Administration (FDA) approved status for internal use in humans. Its degradation occurs via autocatalytic cleavage of the ester bonds through hydrolysis into oligomers and monomers of lactic acid, which are substrates of the Krebs cycle [14]. For this reason, minimal toxicity is usually observed due to its biodegradable and biocompatible properties. Although not toxic, there has been a growing appreciation in immunology of the effects of metabolic byproducts in driving observed immune phenotypes [15,16,17,18,19]. Specifically, lactate has been implicated in modifying inflammatory macrophage responses, although controversy remains as to how lactate acts to do this and whether its role is protective or detrimental [20,21]. Additionally, although PLA is a widely used biomaterial in nanoparticle formulation, its effects following degradation are not well characterized in comparison to other commonly used polymeric materials.

In this study, we assess the physical and biological mechanisms that affect iNP-mediated modulation of macrophage activation by TLR agonists. We hypothesize that the anti-inflammatory effects of iNPs are multimodal, such that the choice of surfactants elicits differences in the nano-bio interactions, while the choice of nanoparticle composition and its degradation products abrogate the activation of proinflammatory cell signaling pathways. Two formulations of iNPs were prepared using PLA with either PVA or PEMA as surfactants to evaluate the role of surface chemistry and charge on inducing anti-inflammatory immune responses. We first evaluated the ability for iNPs to directly interact with PAMPs and the impact of iNP-cell interactions on PAMP-cellular interactions. Next, we assessed the time course-dependent effects of PLA-based iNPs on modulation of NF-κB and p38 mitogen-activated protein kinase (MAPK) signaling. The composition-dependent effects of iNPs on NF-κB and p38 MAPK signaling were subsequently investigated by comparing PLA-based iNPs with commonly utilized commercially available nanoparticles. Lastly, we established a potential role for the pH-sensing G protein-coupled receptor (GPR) 68 on the anti-inflammatory activity of iNPs. Taken together, our study provides evidence for the multimodal mechanisms by which iNPs exert their inherent anti-inflammatory immunomodulatory effects. This work serves as a foundation for further investigation of the inherent immunomodulatory properties of biomaterials and how their specific design features can be tuned to elicit predictable immunological responses through novel strategies and systematic testing with the potential of opening new avenues of research to treat a variety of immune-mediated diseases.

## 2. Materials and Methods

### 2.1. Materials

Acid-terminated PLA of low inherent viscosity in hexafluoro-2-propanol ~0.21 dL/g (approx. 11,700 g/mol) was purchased from Lactel Absorbable Polymers (Birmingham, AL, USA). PEMA (MW 400,000 g/mol) was purchased from Polysciences, Inc. (Warrington, PA, USA). PVA (MW 30,000–70,000 g/mol) was obtained from Sigma-Aldrich (St. Louis, MO, USA). Polystyrene (PS) and poly(methyl methacrylate) (PMMA) particles were purchased from Phosphorex (Hopkinton, MA, USA).

ODN 1668 and ODN 1668 FITC (referred to collectively as CpG ODN) were obtained from Invivogen (San Diego, CA, USA); lipopolysaccharide (LPS) and FITC-conjugated LPS from Escherichia coli serotype O111:B4 were obtained from Sigma-Aldrich (St. Louis, MO, USA).

2X SDS-PAGE sample buffer was produced using 4% SDS; 5.7 M β-mercaptoethanol; 0.2 M Tris-HCl, pH 6.8; 20% glycerol and 5 mM EDTA. RIPA Buffer was purchased from Sigma-Aldrich (St. Louis, MO, USA) and both Halt^®^ Protease Inhibitor Cocktail (100X) and Invitrogen NuPAGE 4–12% Bis-Tris Gel were purchased from Thermo Fisher Scientific (Waltham, MA). Doramapimod (also known as BIRB 796) was purchased from Selleck Chemicals (Houston, TX, USA). Ogremorphin (OGM) was graciously provided by Charles C. Hong [22]. 3-hydroxybutyric acid (3-OBA) was purchased from Sigma-Aldrich.

FITC anti-mouse CD14 mAb (Clone Sa14-2) and PE anti-mouse CD284 (TLR4) mAb (Clone SA15-21) were purchased from BioLegend (San Diego, CA, USA). Phospho-NF-κB p65 (Ser536) (93H1) rabbit mAb, NF-κB p65 (D14E12) XP rabbit mAb, phospho-IκB (Ser32) (14D4) rabbit mAb, IκB (44D4) rabbit mAb, phospho-p38 (Thr180/Tyr182) rabbit Ab, phospho-ERK1/2 (Thr202/Tyr204) (197G2) rabbit mAb, total ERK2 rabbit Ab, phospho-SAPK/JNK (Thr183/Tyr185) rabbit Ab, total SAPK/JNK rabbit Ab, phospho-MKK3 (Ser189)/MKK6 (Ser207) rabbit Ab, MKK6 rabbit Ab, phospho-TAK1 (Thr184/187) rabbit Ab, total TAK1 (D94D7) rabbit mAb, total IRAK4 rabbit Ab, and β-Actin (13E5) rabbit mAb were all purchased from Cell Signaling Technology (Danvers, MA). p38α (C20) rabbit mAb was purchased from Santa Cruz Biotechnology (Dallas, TX, USA). Anti-rabbit IgG (H+L), peroxidase labeled secondary Ab was purchased from Sera Care (Milford, MA, USA).

RAW 264.7 cells were purchased from ATCC (Manassas, VA, USA) and cultured in DMEM (Life Technologies, Carlsbad, CA, USA), penicillin (100 units/mL), streptomycin (100 µg/mL), and 10% heat-inactivated fetal bovine serum (FBS) (VWR, Radnor, PA, USA) at 37 °C and 5% CO_2_.

### 2.2. iNP Preparation and Characterization

PLA iNPs were prepared using the oil-in-water (*o*/*w*) emulsion-solvent evaporation (SE) technique following a similar method as described [10]. Briefly, 200 mg of PLA was dissolved in ethyl acetate at a concentration of 80 mg/mL, 20 mL of 1% PEMA was added then sonicated at 100% amplitude for 30 s using a Cole-Parmer 500-Watt Ultrasonic Homogenizer to make PLA-PEMA. For PLA-PVA, 200 mg of PLA was dissolved in ethyl acetate at a concentration of 300 mg/mL. To this, 5 mL of 2% PVA was added and sonicated at 40% amplitude for 30 s using the same homogenizer. The resulting o/w emulsion was then poured into 100 mL of magnetically stirred 0.5% PEMA (or 0.5% PVA) overnight to remove ethyl acetate. iNPs were then collected by centrifugation at 12,000× *g* for 20 min at 4 °C and washed with 40 mL of MilliQ water. The centrifugation and washing steps were repeated two more times. A mixture of sucrose and mannitol were added to the particle suspension as cryoprotectants to achieve a final concentration of 4% and 3% *w*/*v*, respectively. The nanoparticles were then frozen at −80 °C and lyophilized for at least 48 h prior to use.

The size and zeta potential of all the particles were determined by dynamic light scattering (DLS) using a Malvern Zetasizer ZSP. Cy5.5-labeled PLA particles were prepared by incorporating 1% *w*/*w* of PLA-Cy5.5 into particles as previously described [23].

### 2.3. Particle-TLR Agonist Association Studies

PLA iNPs (concentrations of iNPs as described in the results) were incubated with 1 µg/mL ODN 1668 FITC or 1 µg/mL FITC LPS in sterile DPBS containing 10% heat inactivated fetal bovine serum (FBS). These samples were incubated for 1 h at 37 °C at 5% CO_2_ and vortexed every 10 min. Following incubation, the solutions were centrifuged for 5 min at 12,000× *g* to pellet iNPs then the supernatant was transferred to black 96-well plates to measure fluorescence at 525 nm with the Molecular Devices (San Jose, CA, USA) SpectraMax iD3 Microplate Reader.

### 2.4. Mice

Female C57BL/6J (five to seven weeks old) were purchased from The Jackson Laboratories (Bar Harbor, ME, USA). The mice were housed under specific pathogen-free conditions in a facility at the University of Maryland, Baltimore Veterinary Resources. All mouse procedures and experiments were compliant to the protocols of the University of Maryland, Baltimore Institutional Animal Care and Use Committee (IACUC) and approved under IACUC protocol 0818014.

### 2.5. Isolation and Generation of Bone Marrow-Derived Macrophages (BMMΦs) and Dendritic Cells (BMDCs)

BMMΦs [24] and BMDCs [25] were generated from isolated bone marrow as previously described. Briefly, 5–12-week C57BL/6J female mice were euthanized and the femurs and tibias isolated and flushed with BMMΦ media [RPMI 1640 supplemented with L-glutamine (Life Technologies, Carlsbad, CA), penicillin (100 units/mL), streptomycin (100 µg/mL), 10% heat-inactivated fetal bovine serum (FBS) (VWR, Radnor, PA, USA), and 20% L929 (ATCC) cell-conditioned media] or BMDC media [RPMI 1640 supplemented with L-glutamine, penicillin (100 units/mL), streptomycin (100 µg/mL), 10% FBS, 50 mM β-mercaptoethanol (Sigma-Aldrich) and 20 ng/mL GM-CSF (Peprotech, Rocky Hill, NJ, USA)] using a 1 mL syringe and a 25-gauge needle. Once isolated, the cells were pipetted and filtered through a 40 µm cell strainer then plated in uncoated 10 cm non-tissue culture treated petri dishes. The cells were incubated at 37 °C at 5% CO_2_ and the media was replaced on days 0, 3, 6, and 8. BMMΦs and BMDCs were used for experiments between days 8–10.

### 2.6. Flow Cytometry

Cell staining was conducted according to BioLegend protocols for flow cytometry. Flow cytometry data were collected using a Becton Dickinson LSR II or Becton Dickinson Canto II flow cytometer. Analysis was performed using FCS Express 7 (De Novo Software, Glendale, CA, USA). FcR blocking was performed with the anti-CD16/32 antibody prior to staining. Viability was assessed with 4′,6-diamidino-2-phenylindole dilactate (DAPI) as an exclusion dye for iNP and TLR agonist studies.

### 2.7. Particle-Cell Association Studies

BMMΦs and RAW 264.7 cells were seeded in sterile 24-well plates at a concentration of 0.2 × 10^6^ cells/well and then treated with 30 µg/mL of Cy5.5-labeled iNPs (PLA-PEMA and PLA-PVA) for 1 hr. All treated wells were washed twice with PBS to remove excess iNPs and replenished with 500 µL of fresh sterile PBS. For fluorescence microscopy, cells were either visualized immediately or fixed with 4% paraformaldehyde prior to visualization with either an ECHO (San Diego, CA, USA) Revolve benchtop fluorescence microscope or Nikon (Tokyo, Japan) Eclipse Ti-E confocal microscope. For flow cytometry, cells were scraped using a blunt 1000 µL pipette tip followed by collection by centrifugation and stained for viability using DAPI dye. Flow cytometry was used to measure Cy5.5 signal on viable (DAPI^−^) cells.

### 2.8. Cytokine and Chemokine Secretion Analysis

To evaluate cytokine and chemokine production, BMMΦs were seeded at 0.2 × 10^6^ cells/well in sterile 24-well plates and incubated with 300 µg/mL of the different iNP formulations at 37 °C and 5% CO_2_ for three hours. Excess iNPs were removed by washing twice with PBS followed by replacing with complete medium containing 100 ng/mL LPS or 200 ng/mL CpG ODN. After 48 h, cell culture supernatants were collected and analyzed using enzyme-linked immunosorbent assays (ELISA) (BioLegend) to measure murine interleukin-6 (IL-6) and tumor necrosis factor alpha (TNF-α) or Luminex (Austin, TX, USA) Multi-Analyte Profiling technology (xMAP) to assess multiple cytokines and chemokines as described in the text.

### 2.9. Immunoblotting for Transcriptional Activity

To observe the effects of iNPs on transcriptional activity, BMMΦ were seeded at 1.0 × 10^6^ cells/well in sterile 6-well plates and incubated with 300 µg/mL of the different iNP formulations at 37 °C and 5% CO_2_ for three hours. Excess iNPs were removed by washing twice with PBS followed by replacing with BMMΦ media. Cells were then challenged with 100 ng/mL LPS for 0.5, 1, or 4 h where indicated, at 37 °C and 5% CO_2_ before washing twice with PBS and harvested using 300 µL RIPA buffer containing 1% Halt^®^ Protease Inhibitor. 

Wells treated with BIRB 796 were made to a concentration of 5 µM and incubated for 15 min prior to LPS induction. BIRB 796 was not washed from the wells. Wells treated with OGM were made to a concentration 10 µM and incubated for 4 h at 37 °C and 5% CO_2_, washed twice with PBS to remove excess OGM, replaced with BMMΦ media, followed by iNP treatment as described above. Wells exposed to UV light were exposed in the cell culture hood for 15 min with the plate lids removed. After exposure, media was exchanged and cells were returned to the incubator and harvested at 0.5, 1, or 4 h after exposure.

Protein lysates were generated using 50/50 sample to 2× SDS-PAGE sample buffer. Proteins were then separated by SDS-PAGE and immunoblotted using the antibodies listed above. ECL was used for detection.

### 2.10. Statistical Analyses

Statistical analyses were performed using Prism 9 (GraphPad, San Diego, CA, USA). Results are reported as mean ± standard deviation (SD). A Student’s *t*-test was used to determine the significance of parametric data between groups as labeled. *p* ≤ 0.05 is the cutoff for statistical significance and is denoted throughout the text with *. Additional asterisks are used as applicable to denote the following: ** for *p* ≤ 0.01, *** for *p* ≤ 0.001, and **** for *p* ≤ 0.0001. Comparisons that were not statistically significant were denoted with ns (*p* > 0.05).

## 3. Results

### 3.1. Fabrication, Characterization, and Stability Assessment of Poly(Lactic Acid) iNPs

**Figure 1:**
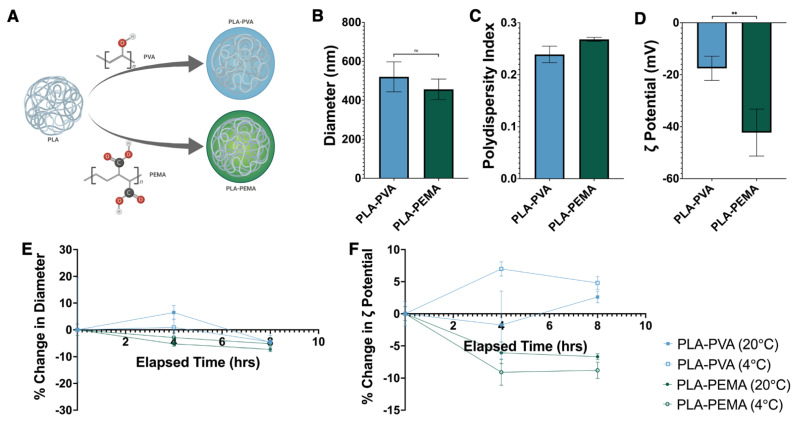
Physicochemical characterization of the synthesized iNPs. (**A**) Schema of the particle formulations utilized for this study. (**B**) Particle diameters were optimized to be in the range of 400–600 nm with (**C**) polydispersity indices in the range of 0.150–0.250. (**D**) Particles were also standardized across surface charge as represented by ζ potential. Additionally, particle stability following reconstitution in distilled water was determined at room temperature (20 °C) and refrigeration (4 °C) over a course of 8 h to confirm stability of particle size (**E**) and zeta potential (**F**). Schematic in (**A**) created with BioRender. Statistical differences between groups were determined by performing Student’s *t*-test. Error bars represent SD. ** for *p* ≤ 0.01 and ns = not significantly different (*p* > 0.05).

iNPs were prepared using PLA by the single emulsion-solvent evaporation method (Figure 1A) with two surfactants—PVA or PEMA. The iNPs produced were similar in size with diameters between 400–600 nm (Figure 1B) with low polydispersity indices (PDI) (Figure 1C). In contrast to size, the zeta potentials of iNPs were significantly different, where PLA-PVA were approximately −17 mV and PLA-PEMA were approximately −40 mV (Figure 1D). We performed additional studies aimed to determine the stability of iNPs following reconstitution in deionized water over 8 h under various storage temperatures [26]. Both PLA-PVA and PLA-PEMA showed less than 10% change in size (Figure 1E). Similarly, the zeta potential of iNPs remained stable with less than 10% variability over 8 h (Figure 1F). Both iNP formulations displayed similar stabilities independent of reconstitution and storage at room temperature or refrigeration.

### 3.2. PLA iNPs Do Not Sequester PAMPs

**Figure 2:**
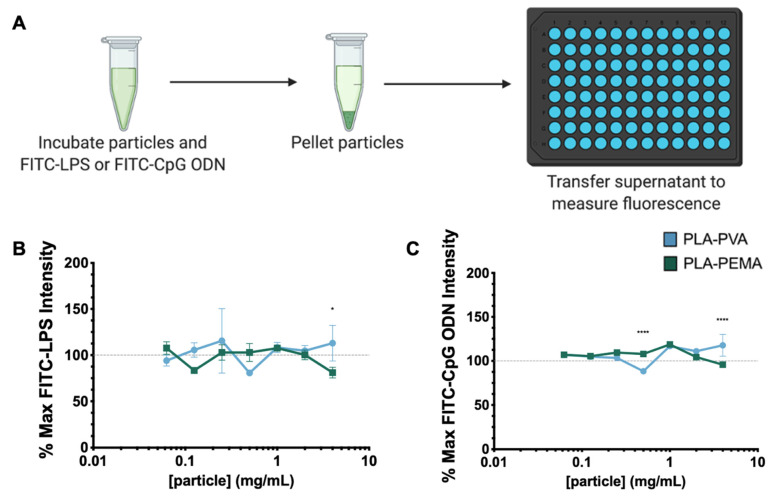
Under typical in vitro serum conditions, both particle types fail to sequester FITC-tagged TLR agonists. (**A**) Particles and FITC-conjugated TLR agonists were co-incubated at 37 °C and 5% CO_2_ for 1 h and then pelleted to determine direct interactions between particles and TLR agonists. When co-incubated with PBS containing 10% FBS, both (**B**) FITC-LPS and (**C**) FITC-CpG ODN fail to interact with particles alone as signified by the dashed line representing 100% FITC signal of FITC-LPS (**B**) or FITC-CpG ODN (**C**) alone. Schematic in (**A**) created with BioRender. Statistical differences between groups were determined by performing Student’s *t*-test. Error bars represent SD. * for *p* ≤ 0.05 and **** for *p* ≤ 0.0001.

One possible mechanism for iNP-mediated anti-inflammatory activity is through functioning as a sink to directly bind PAMPs to sequester them away from TLRs expressed on immune cells [10]. To evaluate the possibility of direct interactions between PAMPs and iNPs (Figure 2A), we incubated PLA-PVA or PLA-PEMA with fluorescein (FITC)-labeled LPS or CpG ODN. Following incubation, the samples were centrifuged to pellet the iNPs and the fluorescence intensity of the supernatant was measured. We tested direct iNP interactions with FITC-LPS and FITC-CpG ODN in PBS containing 10% FBS (Figure 2B,C, respectively). Compared to the FITC-LPS or FITC CpG ODN controls (dashed lines), no concentration-dependent reduction in FITC signal was observed for either iNP tested and the FITC signal variation was less than 20% from the control in all cases. These studies established that iNP sequestration of PAMPs is not a major mechanism by which iNPs elicit their inherent anti-inflammatory effects, warranting further investigation to understand if the protective mechanism is driven directly by iNP interaction with the immune cells of interest.

### 3.3. BMMΦs Associate with and Internalize PLA-PEMA More Extensively Than PLA-PVA

**Figure 3:**
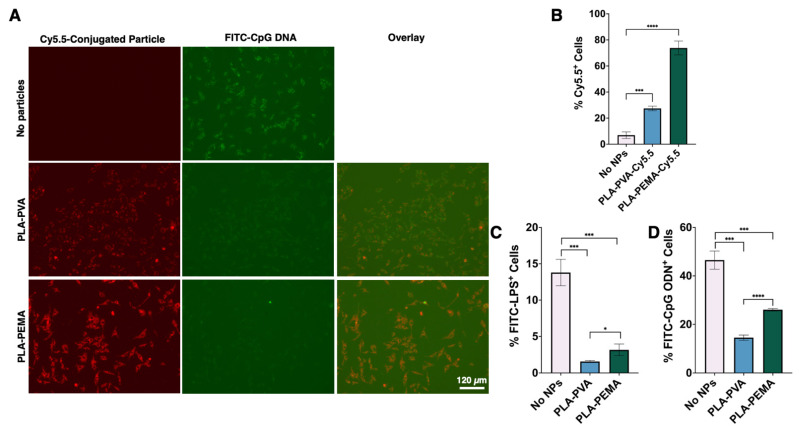
PLA-PVA and PLA-PEMA particle formulations show similar behavior when interacting with TLR agonists and cells with the exception being the increased propensity of PLA-PEMA particle uptake by cells. (**A**) Fluorescence microscopy establishes that PLA-PEMA-Cy5.5 interact to a greater extent with BMMΦs than PLA-PVA-Cy5.5. (**B**) Quantification with flow cytometry after 1-hr co-incubation at 37 °C and 5% CO_2_ confirms cells associate to a greater extent with PLA-PEMA-Cy5.5 particles (30 µg/mL) than PLA-PVA-Cy5.5 particles (30 µg/mL). Both PLA-PVA (300 µg/mL) and PLA-PEMA (300 µg/mL) treatments result in dramatic decreases in BMMΦ cellular uptake of (**C**) FITC-LPS (100 ng/mL) and (**D**) FITC-CpG ODN (100 ng/mL) following 18-hr incubation at 37 °C and 5% CO_2_. Statistical differences between groups were determined by performing a Student’s *t*-test. Error bars represent SD. * for *p* ≤ 0.05, *** for *p* ≤ 0.001, and **** for *p* ≤ 0.0001.

As iNPs do not directly interact with PAMPs, we aimed to further understand the differences in cellular interactions and uptake between various iNPs. To assess iNP-cell interactions, we prepared Cy5.5-conjugated versions of iNPs with similar physicochemical characteristics as unlabeled PLA-PEMA and PLA-PVA (Supplemental Figure S1). Fluorescence microscopy showed that BMMΦs displayed a higher propensity to associate with PLA-PEMA compared to PLA-PVA (Figure 3A), which was also seen using RAW 264.7 cells (Supplemental Figure S2). Flow cytometry was further used to quantitatively measure cell uptake of particles and confirmed that PLA-PEMA associated more rapidly with BMMΦs than PLA-PVA. Within 1 h of iNP incubation with BMMΦs, approximately 75% of BMMΦs were PLA-PEMA-Cy5.5^+^ while only 30% of BMMΦs were PLA-PVA-Cy5.5^+^ (Figure 3B). Since the formulations differed mainly in the surfactant choice and the resultant zeta potential of the iNP, these results suggest that the choice of the negatively charged PEMA drives the propensity of BMMΦs to preferentially interact with iNPs compared to those prepared using PVA.

### 3.4. PLA Particles Hinder LPS and CpG ODN Interaction with BMMΦs

As described above, iNPs do not sequester LPS or CpG ODN (Figure 2), but interact differentially with BMMΦs (Figure 3A,B). Collectively, this suggests that the immunomodulatory activity of iNPs is dependent on their interactions with BMMΦs. To assess this, we first treated BMMΦs with either PLA-PEMA or PLA-PVA followed by incubation with either FITC-LPS (Figure 3C,D) or FITC-CpG ODN (Figure 3A–D). Qualitatively it was observed that despite the greater interaction of PLA-PEMA with BMMΦs, both iNP formulations decreased the association of FITC-CpG ODN with the BMMΦs (Figure 3A). For both LPS and CpG ODN, flow cytometry shows quantitatively that iNP pre-treatment significantly decreased the overall interaction of BMMΦs with LPS and CpG ODN (Figure 3C,D, respectively). These decreases in PAMP interactions with the cells occurs regardless of iNP type, suggesting that iNP-mediated interruption of BMMΦ and LPS or CpG ODN interactions is a process independent of iNP uptake and surfactant composition. Flow cytometry studies (Supplemental Figure S3) revealed reduced CD14 and TLR4 surface molecule expression in response to iNP treatment, suggesting that iNP-mediated disruption of BMMΦ-PAMP interactions may be influenced by the reductions in CD14 and TLR4 surface expression.

### 3.5. PLA-PEMA and PLA-PVA Inhibit NF-κB Activation, But Do So at Different Rates

**Figure 4:**
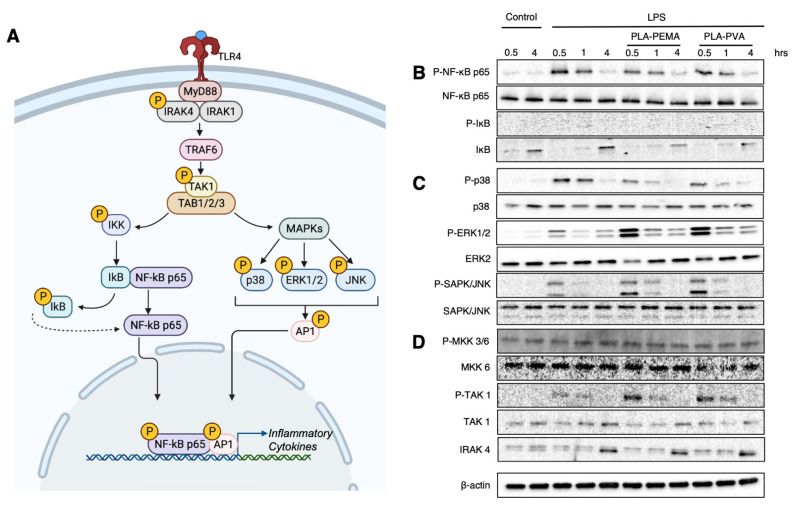
Both iNPs suppress NF-κB p65 and p38 MAPK activation, although at different rates. (**A**) TLR4 activation upon LPS binding triggers a complex signaling cascade where two key nodes of activity involve activation of NF-κB p65 and p38 MAPK to initiate downstream inflammatory cytokine expression. Following PLA-PVA (300 µg/mL) and PLA-PEMA (300 µg/mL) treatment, cells were then stimulated with LPS (100 ng/mL) for 0.5, 1, or 4 h. (**B**) Samples were then immunoblotted for key NF-κB proteins: phospho-NF-κB p65 (Ser536), total NF-κB p65, phospho-IκB (Ser32), and total IκB. (**C**) These samples were also immunoblotted for a variety of MAPKs: phsopho-p38 (Thr180/Tyr182), total p38, phospho-ERK1/2 (Thr202/Tyr204), total ERK2, phospho-SAPK/JNK (Thr183/Tyr185), and total JNK. (**D**) Finally, to address potential impacts of iNPs on upstream signaling proteins, these samples were immunoblotted for phospho-MKK3 (Ser189/)/MKK6 (Ser207), total MKK6, phospho-TAK1 (Thr184/187), total TAK1, IRAK4, and β-Actin. Schematic in (**A**) created with BioRender.

PAMPs engage their respective TLRs and initiate complex signaling cascades that eventually lead to the production of cytokines and chemokines and expression of costimulatory molecules that eventually lead to widespread inflammation [27]. Because these signaling cascades are dependent upon activation of key transcriptional nodes, we next investigated the impact of LPS-mediated TLR4 stimulation via activation of the NF-κB p65 transcription factor and p38 MAPK (Figure 4A). In both signaling pathways, phosphorylation of p65 and p38 signifies engagement and activation of the upstream TLRs. To investigate the effects of iNPs, we first incubated the BMMΦs with iNPs prior to LPS stimulation for 0.5, 1, and 4 h to assess the activation of these key signaling pathways. Figure 4B shows that both PLA-PEMA and PLA-PVA decrease the phosphorylation of p65 compared to no particle treatment. Importantly, the decrease in phosphorylation in the case of PLA-PEMA treatment occurs earlier than that seen in PLA-PVA, suggesting that the more extensive uptake of PLA-PEMA compared to PLA-PVA (Figure 3A,B) plays a role in mediating this protective effect against activation of proinflammatory signaling cascades. Along with this, incubation with either PLA-PEMA or PLA-PVA alone results in no alteration in phosphorylation of either p65 nor a decrease in the total amount of the protein (Supplemental Figure S4). We next probed for MAPKs (Figure 4C). MAPKs are key in that they are activated by different stimuli (including LPS), yet p38, ERK1/2, and SAPK/JNK all have the capacity to phosphorylate transcription factors that form the AP-1 complex, a key regulator of the transcription of inflammatory cytokines [28,29]. We can see that phosphorylation of p38 is decreased secondary to LPS stimulation when treated with iNPs and that this result is opposite to that seen with phospho-ERK1/2 and phospho-JNK. Interestingly, when we evaluate the effects of iNP treatment on MAPK activation alone, we see that iNPs stimulate phosphorylation of ERK1/2, an effect not seen with the other probed MAPKs (Supplemental Figure S4). Finally, an investigation of upstream signaling components shows no decrease in phosphorylation of MKK3, MKK6, and TAK1, nor total levels of IRAK4 suggesting that the iNP-mediated effects downstream of LPS stimulation are limited to NF-κB p65 and p38 MAPK (Figure 4D). These data suggest that the iNP-based modifications to the BMMΦs are inherent to their capacity to respond to an inflammatory trigger rather than some basal change to the BMMΦs.

**Figure 5:**
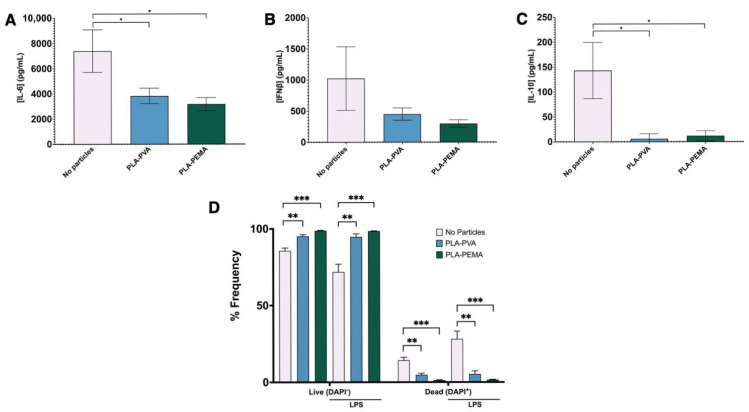
iNP suppression of transcription activation of genes for inflammatory cytokines result in correlated decreases in cytokine production with an increase in BMMΦ survival. Supernatants from cells incubated with LPS for 48 h following iNP treatment were also collected to assess secretion of (**A**) IL-6, (**B**) IFNβ, and (**C**) IL-10, key cytokines produced downstream of TLR4 engagement. (**D**) Additionally, these cells were assessed for viability. Both iNPs, especially PLA-PEMA, result in increased survival based on flow cytometry with DAPI exclusion dye. Statistical differences between groups were determined by performing Student’s *t*-test. Error bars represent SD. * for *p* ≤ 0.05, ** for *p* ≤ 0.01, and *** for *p* ≤ 0.001.

Furthermore, to establish that these transcription changes result in functional changes to the BMMΦs, we used Luminex to establish that these changes in transcription factor activation also resulted in a decrease in cytokine secretions. Indeed, we confirmed this across a multitude of signaling pathways including NF-κB-dependent IL-6 (Figure 5A), IRF3-dependent IFNβ (Figure 5B) [30], and the transcriptionally complex IL-10 (Figure 5C) [31]. Similar experiments conducted with murine macrophage-derived RAW 264.7 cells confirmed iNP-dependent decreases in IL-6 with iNPs following LPS stimulation (Supplemental Figure S5). Interestingly, PLA-PVA treatment resulted in an increase in TNF-α secretion with iNP treatment, while the opposite effect was observed with PLA-PEMA. Additionally, another potential consequence of iNP treatment is the induction of cell death driving the decrease in transcriptional activation and proinflammatory cytokines. We used flow cytometry and cell exclusion dye to establish that our iNPs do not induce cell death and can increase cell survival in the setting of LPS stimulation of BMMΦs (Figure 5D). When taken together, iNPs can drive changes in the function of BMMΦs through reprogramming of transcriptional activation. This leads to decreased proinflammatory cytokine secretions and also aids in extending survival of this cell population.

### 3.6. The PLA Polymer Composition of iNPs Drives the Suppression of NF-κB Signaling

**Figure 6:**
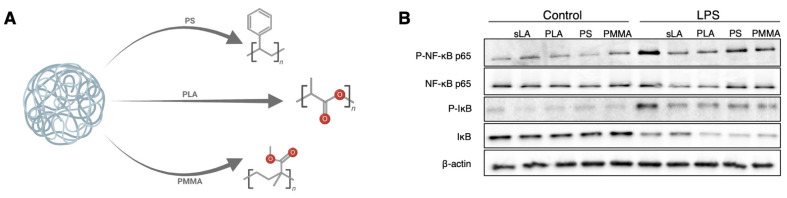
Suppression of NF-κB p65 is dependent upon lactic acid polymer of the particles. (**A**) BMMΦ cells treated with soluble lactic acid (sLA), or nanoparticles composed of PLA-PEMA (PLA), polystyrene-COOH (PS), or poly(methyl methacrylate) (PMMA), for 3 h followed by 1 h LPS stimulation to show differential activation of p65 and greater baseline degradation of IκB compared to sLA and LPS alone following particle incubation and 1h LPS stimulation. (**B**) Samples were immunoblotted for phospho-NF-kB p65 (Ser536), total NF-kB p65, phospho-IκB (Ser32), total IκB, and β-actin. Schematic in (**A**) created with BioRender.

**Figure 7:**
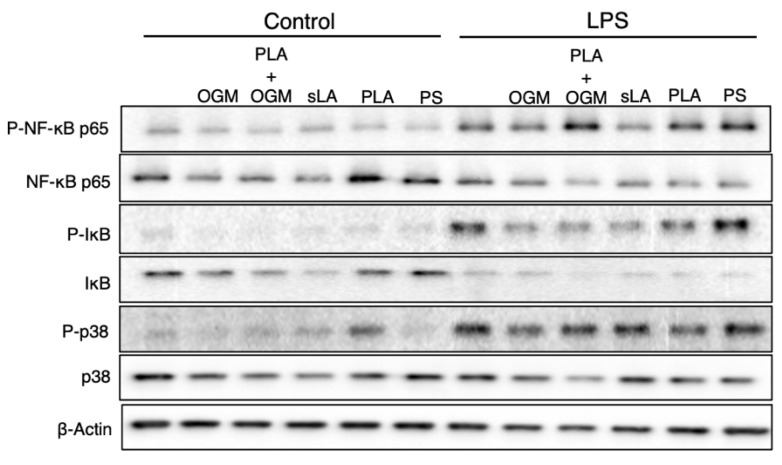
GPR68 inhibition with OGM reverses lactate-mediated suppression of NF-κB p65 activation. BMMΦ cells were treated like previously with soluble lactic acid (sLA) or nanoparticles composed of PLA-PEMA (PLA) or polystyrene-COOH (PS). The addition of OGM, a GPR68 inhibitor, reverses the inhibition of p65 activation seen with PLA following particle incubation and 1-hr LPS stimulation. Samples were immunoblotted for phospho-NF-κB p65 (Ser536), total NF-κB p65, phospho-IκB (Ser32), total IκB, phospho-p38 (Thr180/Tyr182), total p38, and β-actin.

Given that both of our iNP formulations produce an anti-inflammatory immunomodulatory effect, but PLA-PEMA does so more effectively, we next focused our efforts on understanding how these iNPs work by comparing our PLA-PEMA (denoted as PLA in Figure 6 and Figure 7) to commercially available nanoparticle formulations composed of different polymer materials [polystyrene-COOH and poly(methyl methacrylate), herein referred to as PS and PMMA] (Figure 6A). Although both PS and PMMA are non-biodegradable, PS is of particular interest in that it has previously been investigated to be immunomodulatory in studies of inflammatory monocytes via a separate splenic sequestration mechanism [12]. To control for some of the physicochemical properties described as being key to this study (Figure 1), we ensured that the diameter and PDI of the commercial nanoparticles were within range of our iNPs (Supplemental Figure S6). Additionally, because we hypothesize that the lactic acid in our PLA-based iNPs plays a role in mitigating proinflammatory signaling, we also used soluble lactic acid (sLA) as a control to compare its activity to the iNPs. With LPS stimulation (Figure 6B), similarly to sLA, PLA particles suppress NF-κB p65 phosphorylation while PS and PMMA formulations did not, which confirms that the immunomodulatory activity of iNPs was dependent upon the polymers. Of note, when we look at IκB degradation as a marker of NF-κB activation, we see that PLA particles show similar protein levels to PS and PMMA, all of which were lower than for LPS alone and LPS plus sLA. This suggests a PLA-mediated NF-κB suppression unique to the p65 subunit. We then compared the effects of the polymer on mitigating proinflammatory cytokine secretions in response to LPS (Supplementary Figure S7). Again, the PLA-based iNPs successfully suppressed inflammatory cytokine secretion while PS and PMMA showed little immunomodulatory activity as expected based on the NF-κB results.

### 3.7. The Protective Function of the PLA-Based iNPs Depends upon GPR68 Signaling

The inhibition of NF-κB p65 phosphorylation is dependent upon the PLA polymer of our iNPs. We next assessed the mechanism by which the lactic acid from the iNPs elicits its inhibition of inflammatory signaling pathways. Lactic acid is actively removed from the intracellular space [19], therefore we sought to identify the receptor through which the particle-mediated acidity is sensed. Previous work has shown that the GPR68 regulates intestinal inflammation and is a cellular pH sensor [32,33]. We hypothesized that the potential mechanism by which PLA-based iNPs work to inhibit LPS-induced inflammation is through the pH-sensing GPR68. In order to test this, we used OGM [22], a novel inhibitor of GPR68, to block the GPR68-mediated inhibition of inflammation (Figure 7). As expected, PLA iNPs alone showed less NF-κB p65 and p38 MAPK phosphorylation following LPS stimulation than LPS alone or OGM with LPS treatment alone. When we combined both PLA iNPs and OGM with LPS stimulation, not only did OGM increase the level of NF-κB p65 and p38 phosphorylation compared to just PLA iNPs, but it did so to a greater extent than the LPS only control. We further confirmed that the GPR68 inhibitor OGM or GPR81 inhibitor 3-OBA [34] (control) reversed the ability of PLA iNPs to mitigate proinflammatory cytokine secretions (Supplemental Figure S8). These results confirm that inhibition of inflammation is not only mediated by PLA-associated acidification of the microenvironment (negated with OGM) but is specific to sensing of the lactic acid byproduct (inhibited by 3-OBA) of PLA degradation. 

## 4. Discussion

Developing improved treatments for severe inflammation and sepsis is a burgeoning area where nanotechnology-based approaches hold significant promise. Current strategies under development have focused on single-target small molecules and biologics where the failure of these therapeutics in clinical trials suggests a need for strategies with broad activity against proinflammatory immune responses [3,5].

**Figure 8:**
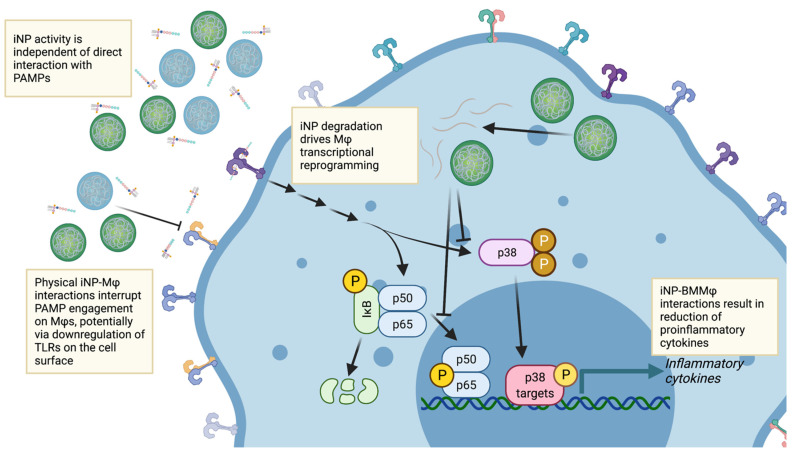
iNPs invoke multiple physical and biological mechanisms to elicit a protective effect in BMMΦs. iNPs interact with BMMΦs to interrupt the engagement of PAMPs on TLRs at both the cell surface and endosomal surface, thus limiting the activation of TLR signaling networks. However, if LPS engages TLR4, the iNPs function via a secondary mechanism whereby their degradation triggers BMMΦ transcriptional reprogramming in response to LPS. This reduces the overall activation and production of inflammation mediators resulting in an overall protective effect to PAMP challenge. Schematic created with BioRender.

iNPs invoke multiple physical and biological mechanisms to accomplish their protective effects (Figure 8). As shown, both types of iNPs lack an ability to directly bind PAMPs including LPS and CpG ODN (Figure 2) but they do alter the ability of BMMΦs to interact with both PAMPs (Figure 3). With this change in BMMΦ-PAMP interaction, it is important to note that although a physical mechanism inhibiting BMMΦ-PAMP interactions is occurring, we cannot yet formally conclude whether cell surface receptor downregulation is the sole response leading to this change (Supplemental Figure S3) or if iNPs serve to directly prevent the interaction of PAMPs with TLRs. Additionally, PLA (but not PLGA) nanoparticles have been shown to downregulate cell surface expression of CD80, CD86, and MHC class II [11,13], suggesting a mechanism by which there is global downregulation of a multitude of key immune cell surface receptors unique to PLA-based nanoparticles. Indeed, engagement of iNPs may trigger endocytosis of these receptors [35], thus making BMMΦs “blind” to PAMP stimulation and perhaps arrested from engaging T cells through the T cell receptor complex [36]. 

Of note, the uptake of iNPs and subsequent cellular transcriptional changes (Figure 4) appear to be independent of this iNP-mediated disruption of BMMΦ-PAMP interaction (Figure 3). Of particular interest is that these iNPs successfully mediate this disruption at two different cellular compartments given that LPS initially binds to TLR4 at the cell surface and TLR4 is then rapidly endocytosed for further LPS binding at the endosomal surface [37,38]. As a further validation of this multi-compartmental activity of iNPs compared to the stimulation of TLR4 at the cell surface prior to endocytosis, TLR9 is endosomal when it is stimulated by CpG ODN [39,40,41]. This suggests that the iNPs serve to disrupt multiple PAMP recognition pathways at different, distinct locations within the cell that lead to proinflammatory cytokine secretion [11], and further emphasizes their potential to serve as a broad-based therapeutic for inflammation. One curiosity that was encountered when evaluating NF-κB- and MAPK-mediated inflammatory signaling is that of all the analytes probed, iNPs drove downregulation of cytokine secretions independent of the formulation; however, TNF-α secretion was increased with PLA-PVA. TNF-α is produced downstream of NF-κB and MAPK activation, but it also has the additional characteristic of further MAPK activation downstream of engagement of its receptor TNFR1 [42]. Additionally, TNF-α exists preformed as pro-TNF-α at the cell membrane until cleavage to the activated form [43], suggesting the possibility that the PLA-PVA iNPs are less effective at inhibiting this cleavage activity.

This proposed mechanism whereby iNPs suppress BMMΦ-PAMP compliments a similar strategy to one employed by Thamphiwatana et al. [44], where macrophage-like NPs served as a sponge for LPS and proinflammatory cytokines. Rather than induce a competition for LPS binding, our experiments show that our iNPs prevented BMMΦ-LPS and BMMΦ-CpG ODN interactions. In combination, these nanoparticle strategies could be combined to further reduce the overall interactions between BMMΦs and stimulating PAMPs. Alone, our iNPs eliminate the need for any cellular material to generate macrophage-like nanoparticles and simplify the synthesis process for the platform since it only requires off-the-shelf chemical components. This potentially avoids regulatory roadblocks in the future with any putative anti-inflammatory therapeutic containing biological components. Additionally, through usage of strictly polymer-based nanoparticles without the need of chemotherapeutic or biologic payloads, we have shown the inherent immunomodulatory capabilities of iNPs that also lend themselves to further modification to suit the needs of other potential therapeutic applications.

As noted, this physical inhibitory iNP activity is assisted by the additional action of reprogramming the functional phenotype of these BMMΦs (Figure 4 and Figure 5). Through alteration of BMMΦ effector activity secondary to LPS challenge, these iNPs take advantage of the inherent plasticity of BMMΦs to modify their activity at the location of PAMP insult. This strategy is of additional benefit in that it serves as a redundant second mechanism at play to synergize with the initial inhibition of BMMΦ-PAMP interactions (Figure 8). Reports of similar nanoparticle-driven innate cell reprogramming has been shown in models of spinal cord injury [45], experimental autoimmune encephalitis [46], and allergic airway inflammation [47]. The culmination of these studies aids in the idea that the iNP-mediated effects on immunomodulation alter the inherent responses of the BMMΦs independent of potential sequestration mechanisms. Given this change in the effector phenotype of the BMMΦs, it remains to be fully elucidated how exactly iNPs elicit these functional responses. Recent work in bone marrow-derived dendritic cells (BMDCs) with PLGA- and PLA-based particles argues that the released lactate from the degradation of these particles lock dendritic cells in an immature phenotype [13,36]. This further suggests that these presumed inert polymeric materials have inherent biological activities that has thus far been under-appreciated, especially the ability of these biomaterials to functionally reprogram the in-situ activity of a variety of immune cells when challenged by known activators of innate immune cells.

Interestingly, these earlier studies and the work described herein highlight the need for increased understanding of the crosstalk between nanoparticle degradation products and the burgeoning field of immunometabolism. PLA is first biodegraded via non-enzymatic random hydrolytic ester cleavage to form oligomers and monomers of lactic acid via surface and bulk erosion [48]. These oligomers and monomers are then free to interact with cells to interact with a variety of cellular processes including the Krebs cycle [14] and, more importantly for our interests, inflammatory pathways. Although degradation of synthetic polymers is better established via passive hydrolysis rather than enzymatic reactions [49], reports in the literature note the existence of fungal [50] and bacterial [50,51] enzymes that can degrade PLA into its monomeric form suggesting a role for infection to drive PLA degradation [52]. Additionally, macrophages and other innate cells secrete an array of enzymes such as lactate dehydrogenase and its coenzyme NADH-reductase during inflammation that can catalyze the degradation of PLA in the setting of PLA implants [53].

When we consider the converse—the role of lactate in modifying the inflammatory response—we see that lactate has been established to play a role in dampening the proinflammatory response within macrophages. An early study compared the role of lactic acid and hydrochloric acid at inducing different inflammatory patterns in RAW 264.7 stimulated with LPS. In this work they showed that when cells were titrated to more acidic environments such as pH 6.5, HCl treatment essentially drove a proinflammatory response with LPS stimulation as measured by evolution of NO, IL-6-to-IL-10, and NF-κB DNA binding. In contrast, lactic acid treatment (controlled for pH) effectively inhibited LPS-induced NO, IL-6, IL-10, and NF-κB DNA binding [54]. This work is key because it establishes that the acidity of the environment alone does not alone drive the anti-inflammatory effects that we have also observed, but rather that lactate serves as a unique molecule driving the suppression of inflammatory responses in macrophages. Further work built upon this to establish a key role for GPR81, a cell-surface receptor for lactate, in mediating lactate suppression of proinflammatory responses in the GI tract using animal models for dextran sulfate-sodium-induced colitis [55] and acute hepatitis and pancreatitis [56]. Interestingly, in other inflammatory models utilizing macrophages from non-GI sources, the role of GPR81 in lactate-mediated responses remains controversial [20,21] suggesting the potential of other pH-sensing receptors, such as GPR68, to play a complementary role [57].

## 5. Conclusions

Taken together, this work establishes that iNPs take advantage of multiple mechanisms to mitigate severe inflammatory responses and suggests a novel multimodal approach to improve prospects for patients with sepsis and other inflammation-mediated diseases. Polymer-based nanoparticles show promise in serving as drug carriers for controlled delivery of active chemotherapeutic agents; however, the inherent immunomodulatory nature of the materials themselves remains not well characterized. We have described the nano-bio interactions for PLA-based iNPs with varying surface charge and applied these formulations to modulating BMMΦ activity in response to diverse inflammatory agents. We showed that iNPs modify proinflammatory cytokine secretions and also establish that the mechanisms by which this occurs are broad and rely on both physical interactions and reprogramming of BMMΦs. Physical interaction of the BMMΦs with iNPs limit uptake of LPS and CpG ODN interaction. Furthermore, iNPs elicit intrinsic changes in the BMMΦs through metabolic alterations such that NF-κB and p38 MAPK activity is downregulated in response to LPS stimulation. Future studies aim to address applications of iNPs to improve clinical outcomes in murine models of severe inflammation and sepsis and to further characterize nano-bio interactions of iNPs with other key players of the innate immune response, particularly those regulating immunometabolism.

## Data Availability

The data presented in this study are available upon request from the corresponding author, R.M.P., upon request.

## Supplementary Materials

The following are available online at https://www.mdpi.com/article/10.3390/pharmaceutics13111841/s1, Figure S1: Physicochemical characterization of the iNPs with their fluorophore-conjugated formulations, Figure S2: Confocal microscopy showing extent of iNP uptake in RAW 264.7 cells and subsequent colocalization with FITC-CpG ODN, Figure S3: Flow cytometry reveals iNPs trigger downregulation of CD14 and TLR4 surface expression in bone marrow-derived dendritic cells, Figure S4: Control immunoblots showing p38 inhibition with BIRB 796 and lack of NF-κB p65 and p38 MAPK activation with iNPs alone, Figure S5: LPS-stimulated RAW 264.7 IL-6 and TNF-α secretion using the same experimental design as in Figure 4, Figure S6: Physicochemical characterization of commercially available particles, Figure S7: LPS-stimulated RAW 264.7 IL-6 and TNF-α secretion using the same experimental design as described previously with the inclusion of commercially available particles as described in Figure 5, Figure S8: LPS-stimulated RAW 264.7 IL-6 and TNF-α secretion using the same experimental design as described previously with the inclusion of GPR68 and GPR81 inhibitors described in Figure 6.
