# Supplementary material for: Immunomodulatory Nanoparticles Mitigate Macrophage Inflammation via Inhibition of PAMP Interactions and Lactate-Mediated Functional Reprogramming of NF-κB and p38 MAPK
Source: Pharmaceutics. 2021 Nov 2;13(11):1841. doi: 10.3390/pharmaceutics13111841 (PMC8618039; doi:10.3390/pharmaceutics13111841)
Supplement: Supplementary file 1 [file pharmaceutics-13-01841-s001.zip › pharmaceutics-1388183-supplementary.pdf]

pharmaceutics

MDPI

Supplementary Materials: Immunomodulatory Nanoparticles Mitigate Macrophage Inflammation via Inhibition of PAMP Interactions and Lactate-Mediated Functional Reprogramming of NF-κB and p38 MAPK

Jackline Joy Martín Lasola, Andrea L. Cottingham, Brianna L. Scotland, Nhu Truong, Charles C. Hong, Paul Shapiro and Ryan M. Pearson

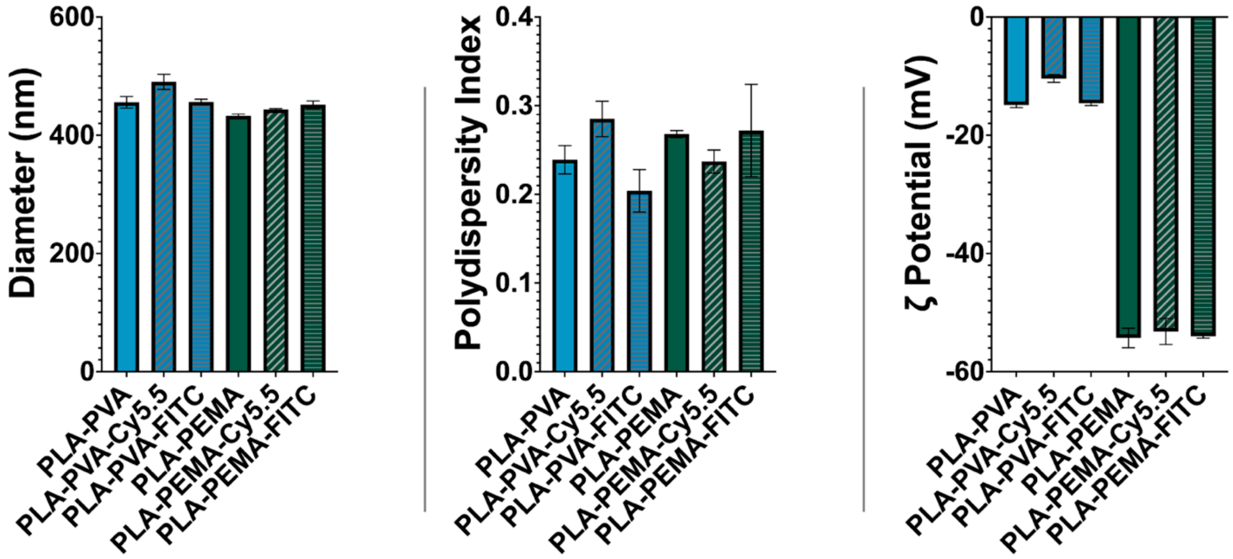

Figure S1. Physicochemical characterization of the iNPs with their fluorophore-conjugated iNP formulations.

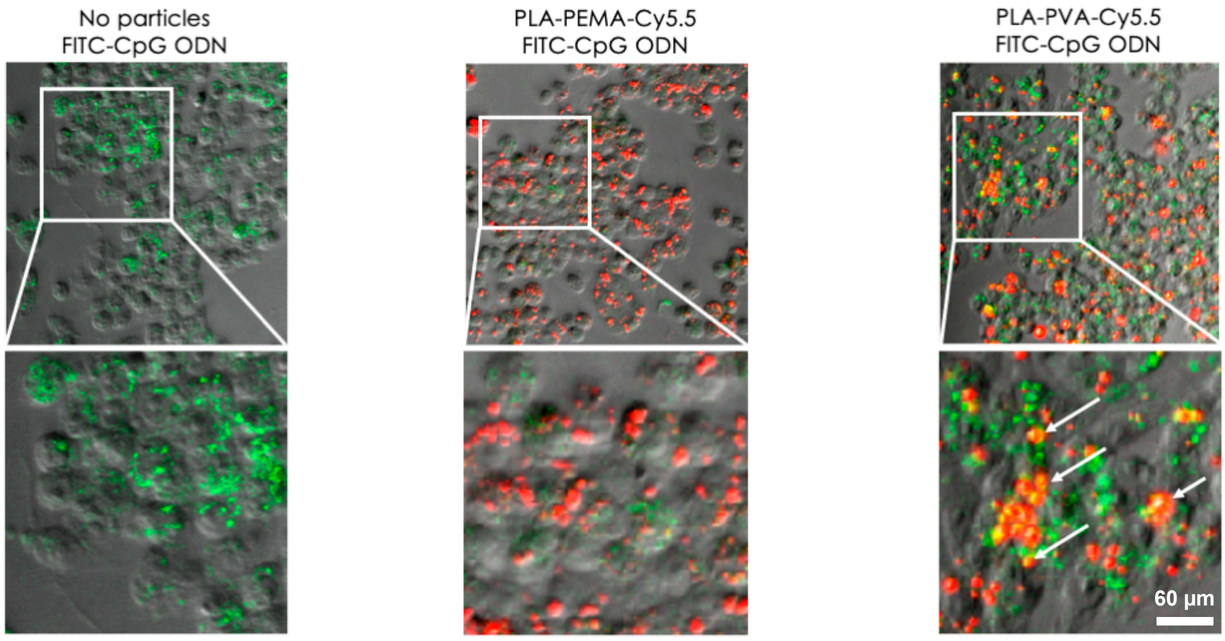

Figure S2. Confocal microscopy showing extent of iNP uptake in RAW 264.7 cells and subsequent colocalization with FITC-CpG ODN.

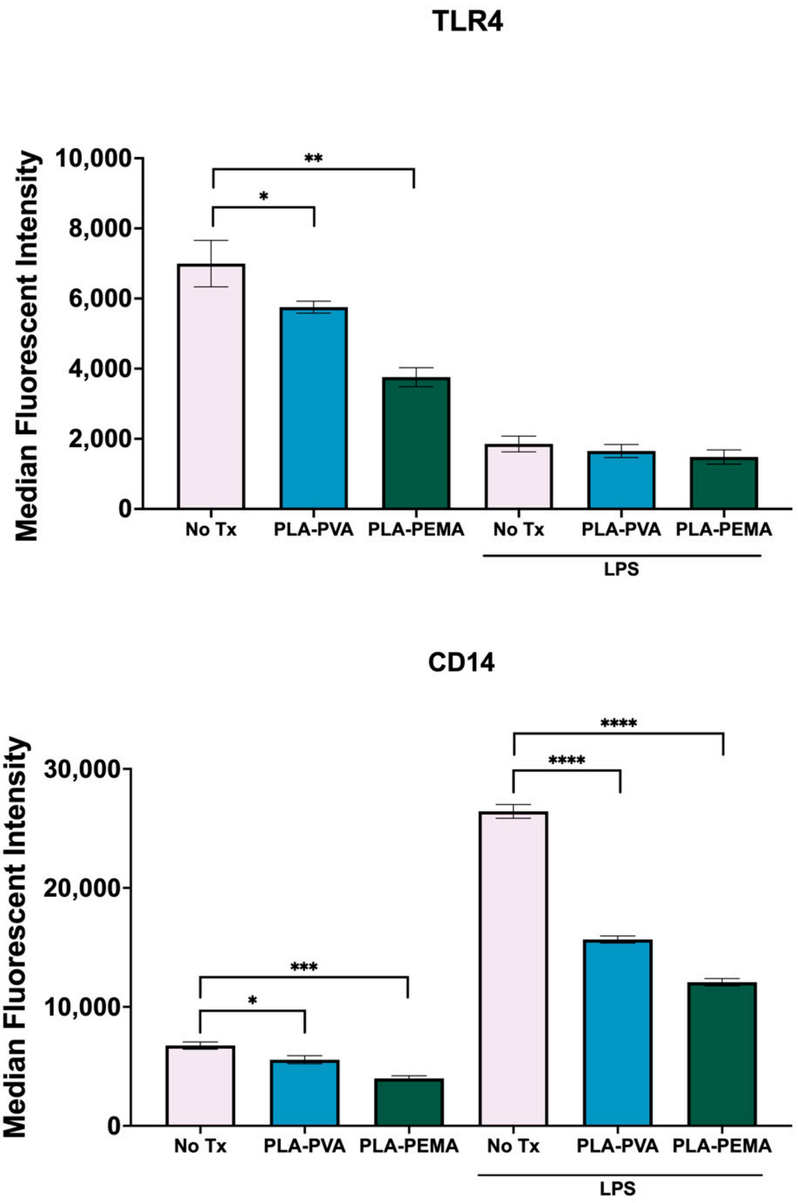

Figure S3. Flow cytometry reveals iNPs trigger downregulation of TLR4 and CD14 surface expression in bone marrow-derived dendritic cells. Statistical differences between groups were determined by performing Student's t-test. Error bars represent SD. * for $p \leq 0.05$, ** for $p \leq 0.01$, *** for $p \leq 0.001$, and **** for $p \leq 0.0001$.

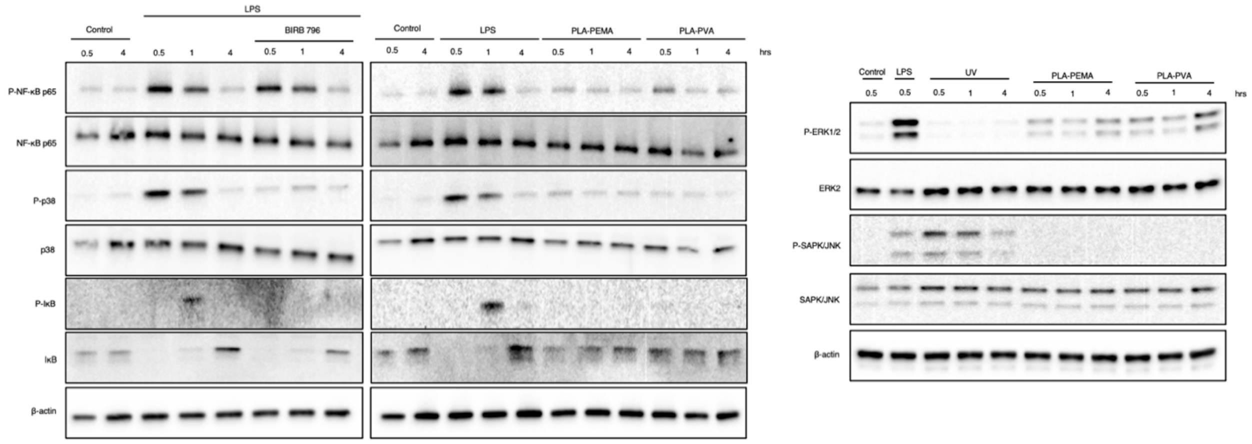

Figure S4. Control immunoblots showing p38 inhibition with BIRB 796 and lack of NF-κB p65 and p38 MAPK activation with iNPs alone. Interestingly, note that ERK1/2 is phosphorylated under iNP treatment to a greater extent than that found at baseline (control at 0.5 hrs). There is, like p38, no activation of the SAPK/JNK MAPK with iNPs alone with LPS or UV treatment of cells as the positive control. Associated with figure 4.

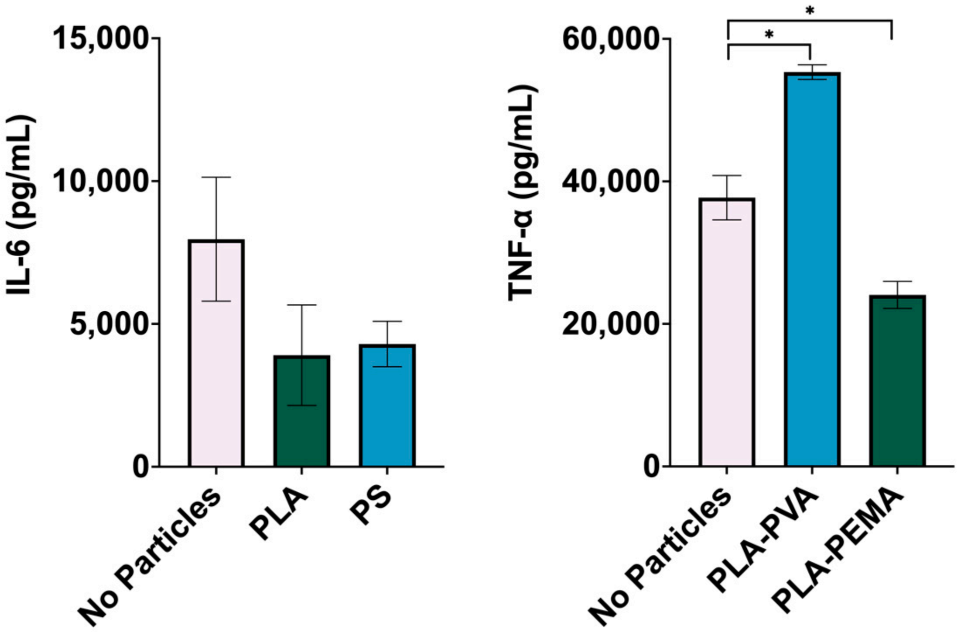

Figure S5. LPS-stimulated RAW 264.7 IL-6 and TNF-α secretion using the same experimental design as in figure 4. Like the results seen with BMMΦs, we see the same abrogation of IL-6 secretion with RAW 264.7 cells. Interestingly, TNF-α secretion shows a differential response with the expected abrogation seen with PLA-PEMA an opposite effect seen with PLA-PVA. Statistical differences between groups were determined by performing Student's t-test. Error bars represent SD. * for $p \leq 0.05$.

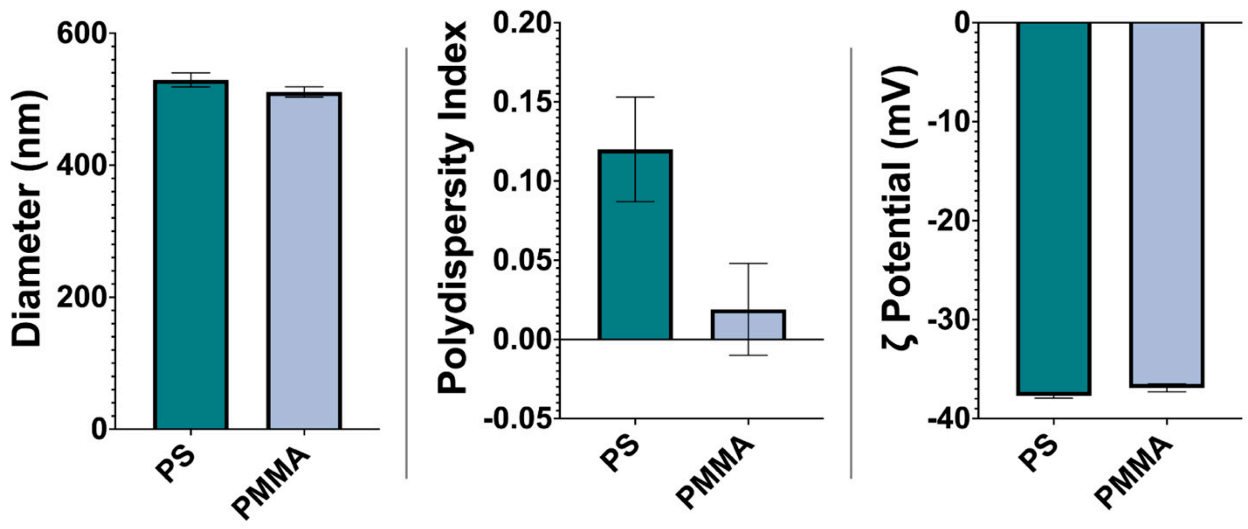

Figure S6. Physicochemical characterization of commercially available particles. PS: polystyrene; PMMA: poly(methyl methacrylate).

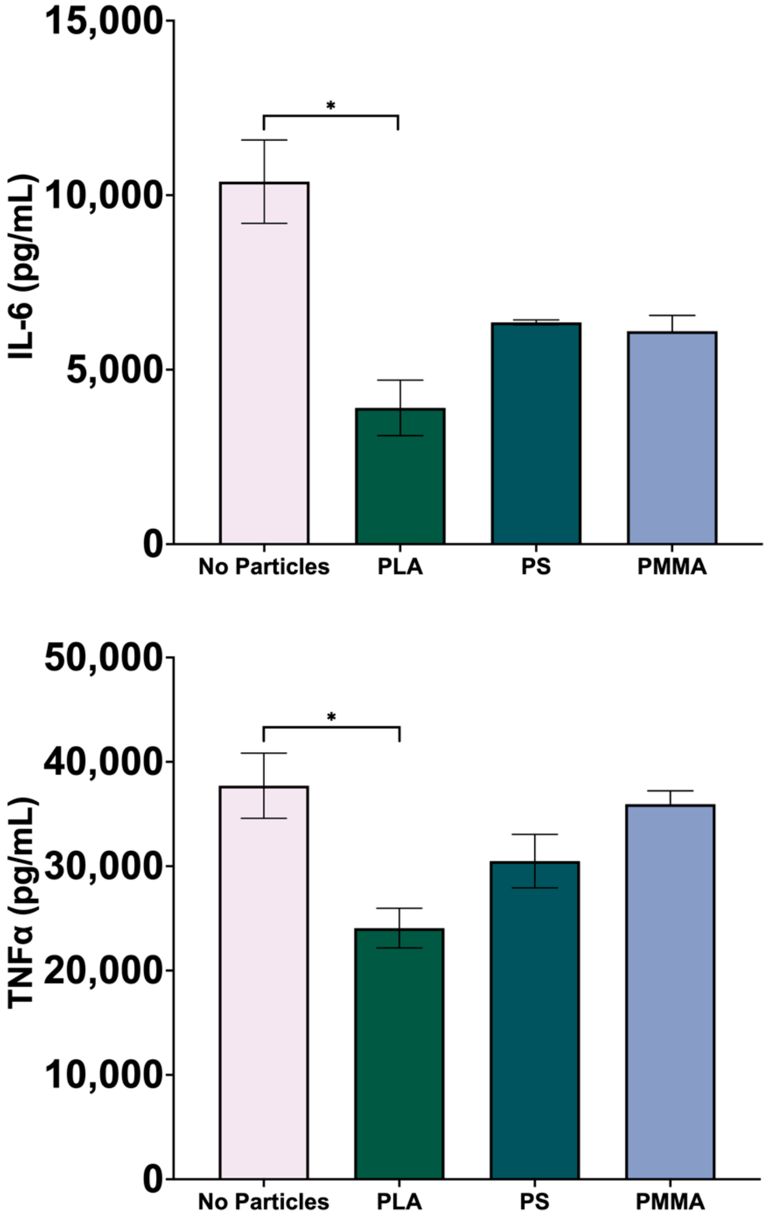

Figure S7. LPS-stimulated RAW 264.7 IL-6 and TNF-α secretion using the same experimental design as described previously with the inclusion of commercially available particles as described in figure 5. Statistical differences between groups were determined by performing Student's t-test. Error bars represent SD. Error bars represent SD. * for $p \leq 0.05$.

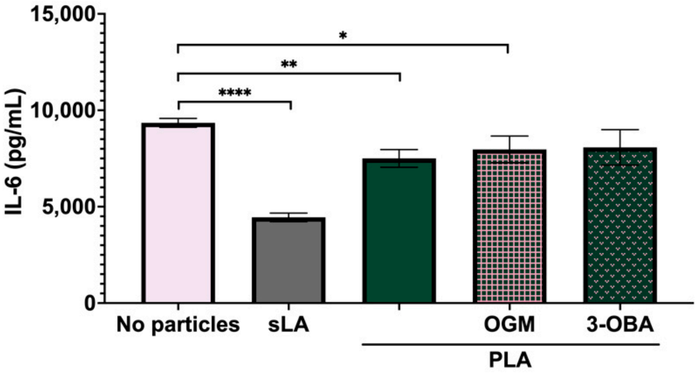

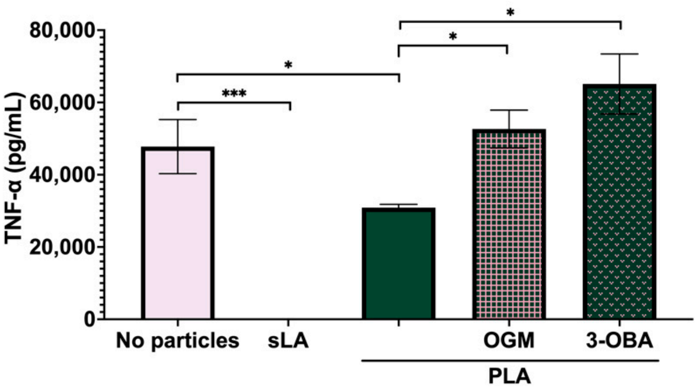

Figure S8. LPS-stimulated RAW 264.7 IL-6 and TNF-α secretion using the same experimental design as described previously with the inclusion of pH and lactate receptor inhibitors described in figure 6. Statistical differences between groups were determined by performing Student's t-test. Error bars represent SD. * for $p \leq 0.05$, ** for $p \leq 0.01$, *** for $p \leq 0.001$, and **** for $p \leq 0.0001$.
